# Intrathoracic tuberculous lymphadenopathy in children: a guide to chest radiography

**DOI:** 10.1007/s00247-017-3890-1

**Published:** 2017-08-29

**Authors:** Anthony George, Savvas Andronikou, Tanyia Pillay, Pierre Goussard, Heather J. Zar

**Affiliations:** 1 Department of Paediatric Radiology, Bristol Royal Hospital for Children and the University of Bristol, Paul O’Gorman Building, Upper Maudlin St., Bristol, BS2 8BJ UK; 2 Department of Paediatrics and Child Health, Red Cross War Memorial Children’s Hospital, University of Cape Town and Medical Research Council Unit on Child and Adolescent Health, Cape Town, South Africa; 3 Department of Paediatrics and Child Health, Tygerberg Hospital and the University of Stellenbosch, Cape Town, South Africa

**Keywords:** Chest radiography, Children, Computed tomography, Lymph nodes, Magnetic resonance imaging, Pulmonary tuberculosis, Standardisation

## Abstract

Making the diagnosis of pulmonary tuberculosis in children can be difficult because microbiological confirmation is not often achieved. Diagnosis is therefore often based on clinical features in combination with chest radiograph findings. Chest radiographs can demonstrate lymphadenopathy of the hilar and para-tracheal regions on the anteroposterior view, and subcarinal lymphadenopathy on the lateral view. However poor interobserver agreement has been reported for radiologist and clinician assessment of lymphadenopathy. This might reflect the lack of standardised imaging criteria for diagnosis as well as radiologists’ objectives for achieving sensitivity rather than specificity. In this paper the authors provide a pictorial aid of chest radiographs in children with culture-confirmed tuberculosis to help clinicians identify lymph node enlargement in primary pulmonary tuberculosis. This collection of images comprises chest radiographs accompanied by schematics and either CT or MRI scan confirmation of pathological lymph node enlargement at the positions commonly affected in tuberculosis.

## Introduction

Making the diagnosis of pulmonary tuberculosis in children can be difficult because microbiological confirmation is not often achieved [1]. Diagnosis is therefore often based on clinical features in combination with chest radiograph findings. Recently, imaging diagnosis using CT, mediastinal US and MRI has been proposed, but the chest radiograph remains the most frequently used diagnostic imaging tool in children because it is readily available in most clinical settings [1]. With any of the imaging techniques, identification of lymphadenopathy is the major sign for diagnosing paediatric pulmonary tuberculosis [2]. However tuberculous lymphadenopathy can be difficult to diagnose with confidence on chest radiographs [3], and significant inter- and intra-observer variability has been reported for clinicians interpreting these (average weighted kappa =0.33) [4]. As a result, several chest radiograph classification systems have been devised to aid the diagnosis of pulmonary tuberculosis, and these involve assessment of the lung parenchyma and presence of intrathoracic lymphadenopathy [5, 6]. However these have not been widely included in routine use because they fail to provide adequate radiologic criteria, have not been well validated and are not backed up with a standard set of images [7].

Despite their many limitations, chest radiographs demonstrate lymphadenopathy of significant size at the hilar and para-tracheal regions on the anteroposterior view (as asymmetrical lobulated soft-tissue masses, often with sharply defined margins) and in the subcarinal region on the lateral view [2, 8]. Enlarged tuberculous lymph nodes can compress and displace the airways, offering further indirect clues for diagnosis [2, 5, 8]. Despite the known and reported features of lymphadenopathy in children with pulmonary tuberculosis, wide inter-observer variability has been reported for radiologists and clinicians identifying these on chest radiographs [4]. This might reflect the lack of standardised imaging criteria for diagnosis [7] as well as radiologists’ perceived objectives of achieving sensitivity rather than specificity [9].

CT is considered the modality of choice for identifying mediastinal and hilar lymphadenopathy [2, 10] and can demonstrate these in children with normal or equivocal chest radiographs. CT not only demonstrates lymphadenopathy to greater advantage, but it also demonstrates any calcification, parenchymal disease or complication including airway compression, air-trapping and pleural disease [10], which adds confidence for the reader. CT can therefore act as a gold standard for the presence of lymphadenopathy in children with pulmonary tuberculosis. MRI can confirm the presence of hilar and mediastinal lymphadenopathy. MRI is well-established for imaging lymphadenopathy in thoracic lymphoma, and there are also reports of MRI use for diagnosing tuberculosis and other lung infections [11, 12].

In the context of standardising the interpretation of chest radiographs of children with suspected pulmonary tuberculosis in clinical practice, it can be useful to have a pictorial aid of chest radiographic images in children with culture-confirmed tuberculosis and definite lymphadenopathy confirmed through cross-sectional imaging. This could act in a similar manner to the templates of images used in other areas of diagnostic imaging where standardisation of imaging features for diagnosis is vital, such as asbestosis scoring on chest radiograph [13] and ovarian cyst classification on US [14]. Physicians and allied staff interpreting chest radiographs in children with suspected pulmonary tuberculosis could refer to the images and schematics provided in this pictorial review regarding definite signs of lymphadenopathy when trying to improve specificity. To that end, the images used in this review illustrate lymph node enlargement in typical positions associated with primary pulmonary tuberculosis in children with culture-confirmed tuberculosis.

## Normal anteroposterior chest radiographs in children

**Fig. 1:**
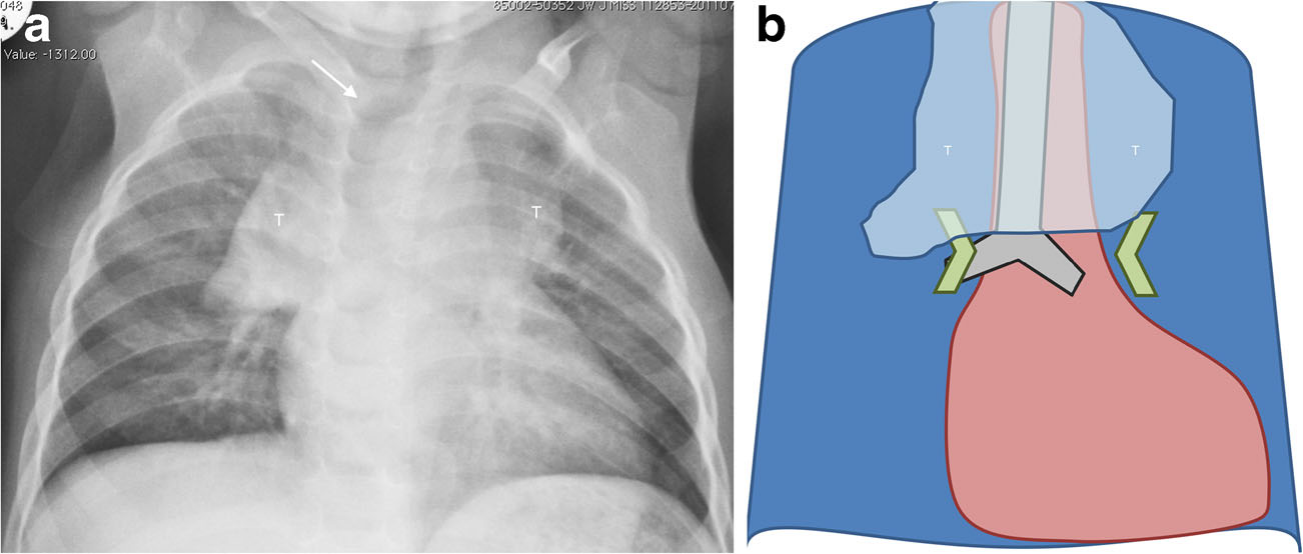
Normal chest radiography in a 14-month-old girl. **a**, **b** Anteroposterior chest radiograph (**a**) and accompanying schematic (**b**) demonstrate normal appearance with physiological buckling of the trachea towards the right (*arrow* in **a**) and the width of the mediastinum contributed to by the thymus (*T*)

Figure 1 shows the normal appearances of a paediatric chest on anteroposterior chest radiographs. There are several features to assess on a radiograph performed to detect enlarged lymph nodes. The normal trachea should either be displaced to the right by the presence of a normal left-side aortic arch or should be at least central. The normal thymus and heart are relatively large in young children [8] and therefore the mediastinal width and para-tracheal soft-tissue thickness are not parameters that should be evaluated in the detection of mediastinal lymphadenopathy in the younger age groups. It is important to note, however, that the normal thymus does not compress or displace any structures and that it is a soft structure that itself can be compressed. The hilar points (the apparent intersections of the lowest upper-lobe pulmonary veins and the lower-lobe pulmonary arteries) should have a clear outward V-shape configuration, i.e. the hilar points must be visible, should always be *concave* outwards and the space between the main intersecting vessels should be empty of soft-tissue masses. There should be no compression of the major bronchi.

## Right hilar lymphadenopathy

**Fig. 2:**
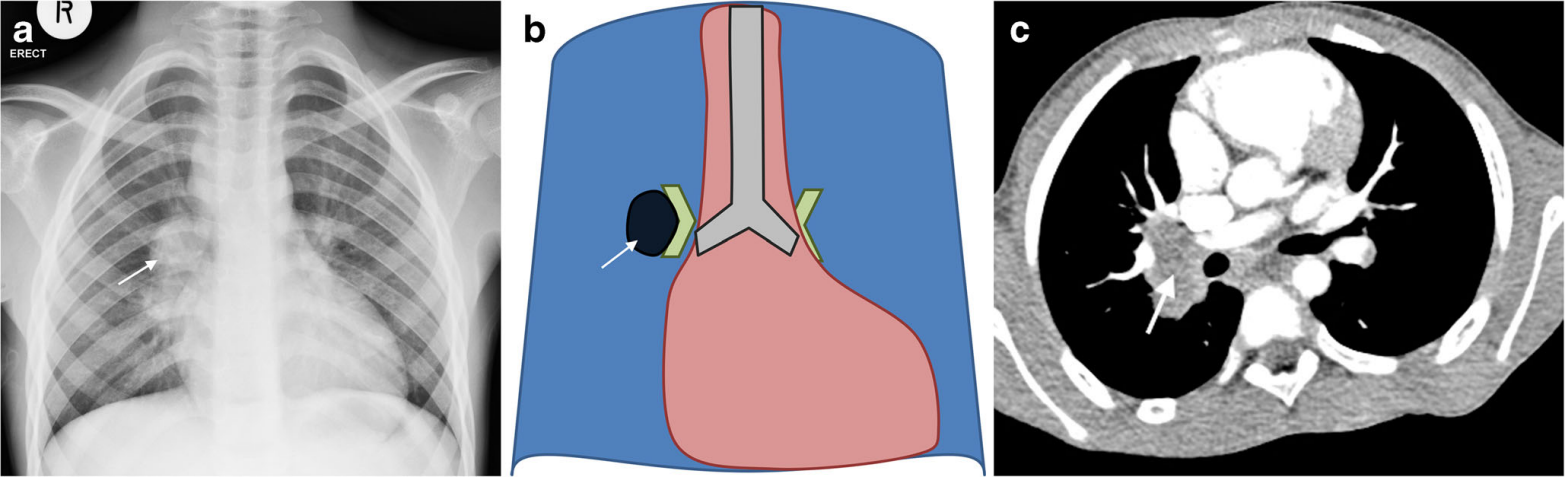
Right hilar lymphadenopathy and culture-confirmed tuberculosis in a 9-month-old girl. **a**–**c** Anteroposterior chest radiograph (**a**), schematic (**b**) and accompanying axial post-contrast CT (**c**) demonstrate filling of the hilar point by a dense soft-tissue mass, resulting in an outwardly convex outline (*arrow*) as opposed to the expected concavity of the converging vessels

Right hilar lymphadenopathy is seen on the anteroposterior radiograph as a lobulated density occupying the hilum and obliterating the hilar point (which should normally be a crisp V-shape meeting of large vessels), resulting in an outwardly *convex* appearance [2]. Figure 2 shows the characteristic features of right hilar lymphadenopathy on chest radiograph. CT confirms that non-enhancing lymphadenopathy occupies the right hilar position.

## Right paratracheal lymphadenopathy

**Fig. 3:**
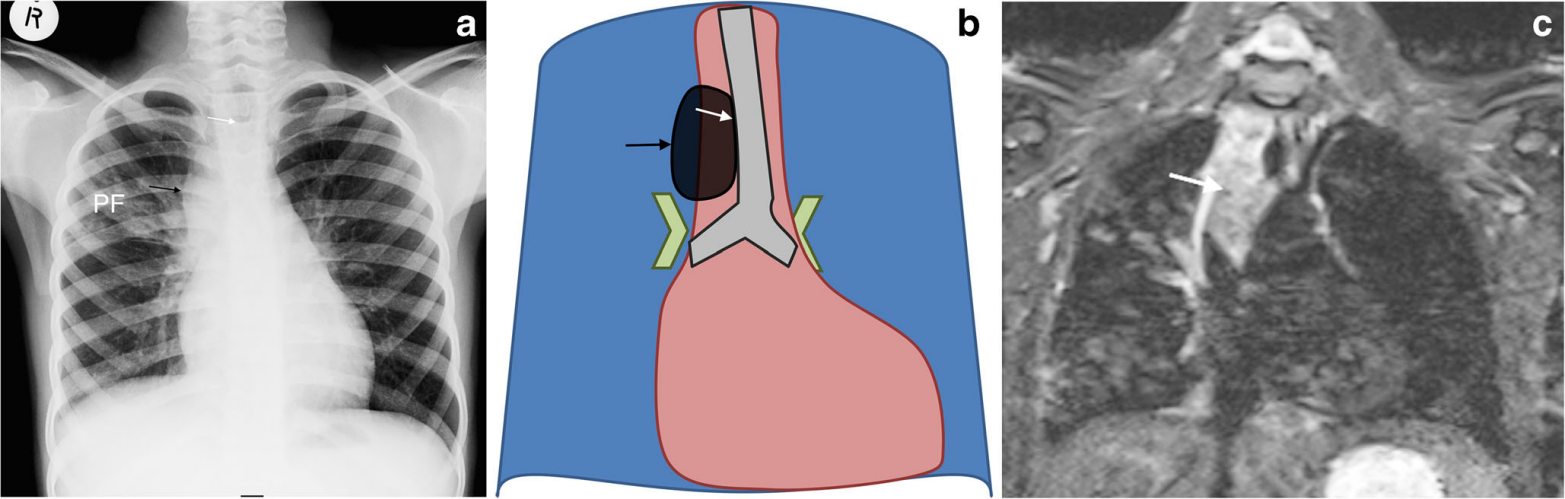
Right paratracheal lymphadenopathy and culture-confirmed tuberculosis in an 8-year-old boy. **a**–**c** Anteroposterior chest radiograph (**a**), schematic (**b**) and accompanying coronal short tau inversion recovery MRI (**c**) demonstrate characteristic features of right paratracheal lymphadenopathy (*black arrow* in **a**, **b**) in addition to nodular parenchymal disease in the right upper zone, the primary focus (*PF* in **a**). The mid trachea (*white arrow*) is bowed, convex to the left and displaced to the left. The MRI (**c**) confirms the absence of normal thymic tissue in the area of interest

Because of the presence of a thymus and other normal structures making up the mediastinum, lymphadenopathy superimposed on these can be masked on the anteroposterior radiograph [2]. Lymphadenopathy should only be reported if there are clear lobulated paratracheal soft-tissue masses extending beyond the thymic and cardiac margins, with airway compression or displacement [8]. Consideration of the position, contour and caliber of the trachea is therefore extremely useful for confirming the presence of mediastinal lymphadenopathy on the anteroposterior radiograph [2]. Figure 3 demonstrates characteristic features of right paratracheal lymphadenopathy in addition to nodular parenchymal disease in the right upper zone (the primary focus). The mid trachea is bowed, convex towards and displaced to the left. The accompanying short tau inversion recovery MRI confirms a low signal intensity mottled lymph node complex on the right side of the superior mediastinum and demonstrates that there is no normal thymus present.

## Left hilar lymphadenopathy

**Fig. 4:**
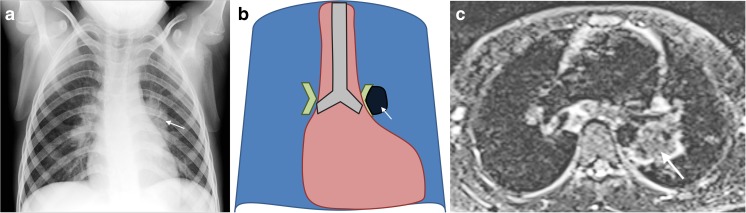
Left hilar lymphadenopathy and culture-confirmed tuberculosis in a 6-year-old boy. **a**–**c** Anteroposterior chest radiograph (**a**), schematic (**b**) and axial short tau inversion recovery MRI (**c**) demonstrate typical features of left hilar lymphadenopathy. There is a rounded increased-density soft-tissue mass with a convex lateral border projected beyond the upper left cardiac silhouette (*arrow*) and there is loss of the clear V-shape hilar point

Left hilar lymphadenopathy is reported less often in children because it only becomes obvious on anteroposterior chest radiographs when it projects beyond the left cardiac margin [8]. Left hilar lymphadenopathy might also be evident on the anteroposterior chest radiograph when a child is rotated to the right, but this is at the expense of not seeing the right hilum clearly. Sometimes left hilar lymphadenopathy is seen through the heart shadow as a dense lobulated soft-tissue mass. Figure 4 demonstrates the typical features of left hilar lymphadenopathy. There is a rounded, increased-density soft-tissue mass with a convex lateral border projected beyond the upper left cardiac silhouette and there is loss of the clear V-shape hilar point. Short tau inversion recovery MRI can confirm the presence of low-signal-intensity hilar lymphadenopathy occupying the left hilum.

## Left paratracheal lymphadenopathy

**Fig. 5:**
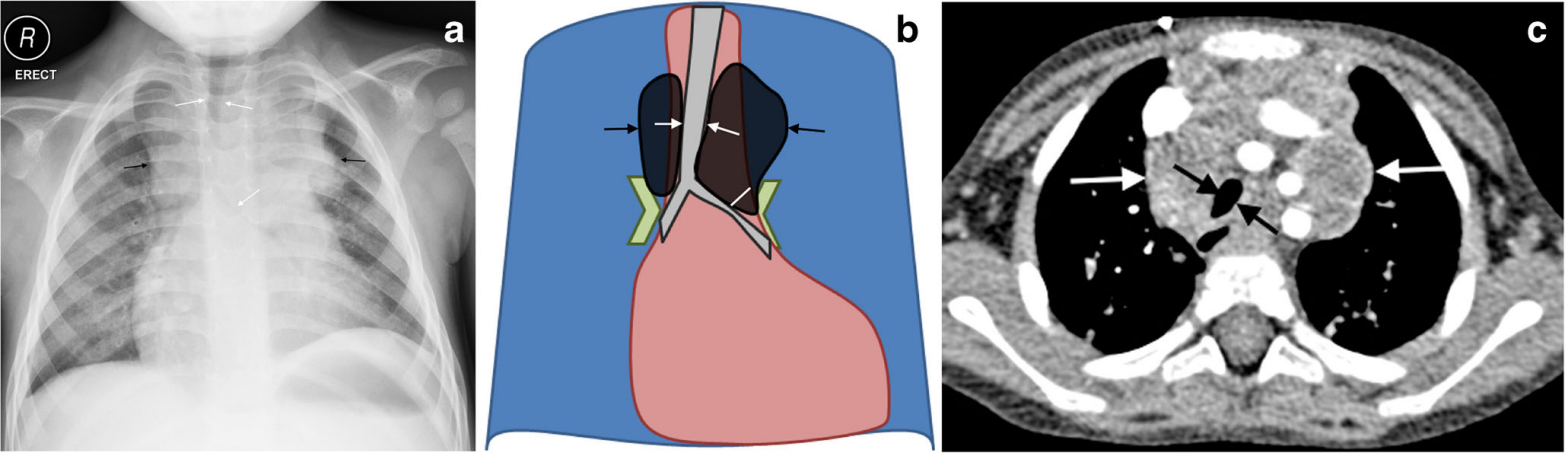
Bilateral paratracheal lymphadenopathy and culture-confirmed tuberculosis a 1-year-old boy. **a**–**c** Anteroposterior chest radiograph (**a**), and schematic (**b**) demonstrate bilateral paratracheal lymph node enlargement (*black arrows*). It can be assumed that the left superior mediastinal mass is not made up of the thymus because the trachea and left main bronchus are compressed (*white arrows*). On CT (**c**) the enlarged lymph nodes are of low attenuation with a rim of enhancement, typical for centrally necrotic tuberculous lymphadenopathy, white arrows and the trachea is compressed black arrows

Left paratracheal lymphadenopathy is not often identified in isolation and is usually reported when the whole anterior mediastinum is involved. Figure 5 shows both left and right paratracheal lymph node enlargement on the anteroposterior chest radiograph. It can be suspected that the left superior mediastinal mass is not from the normal thymus when the left main bronchus is depressed and compressed. Airway compression is an ancillary sign of intra-thoracic lymphadenopathy, seen more often in infants than older children [5]. On CT, the lymph nodes show low attenuation with a rim of enhancement, typical for tuberculous adenopathy, which is necrotic centrally [2, 8].

## Airway compressions and multifocal disease

**Fig. 6:**
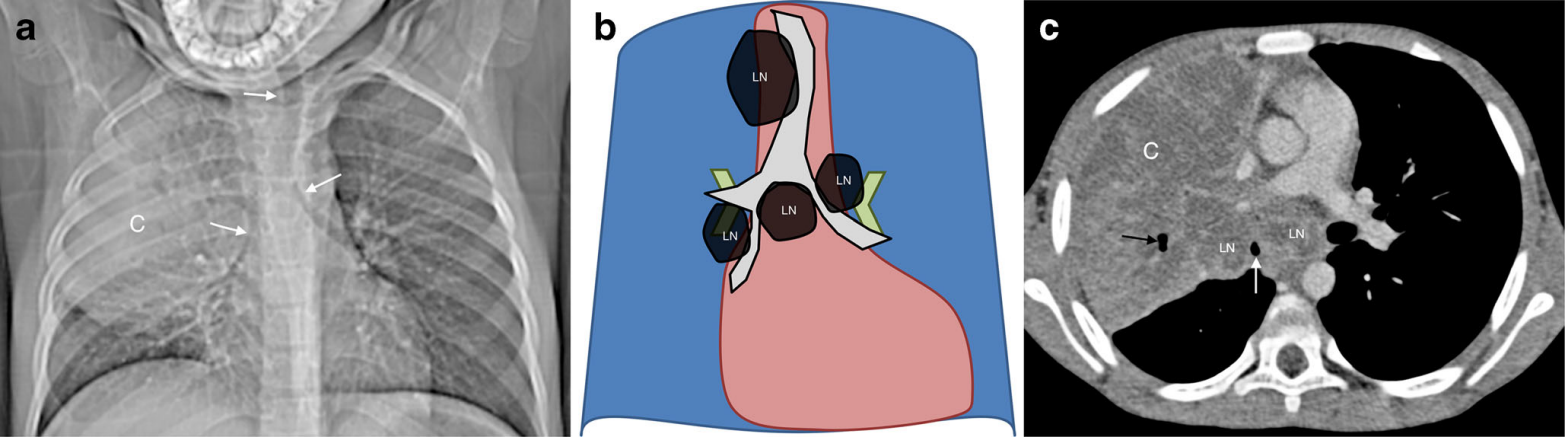
Multifocal lymphadenopathy and airway compression and culture-confirmed tuberculosis in an 8-month-old boy. **a**–**c** Anteroposterior chest radiograph (**a**), schematic (**b**) and axial post-contrast CT (**c**) demonstrate multifocal nodal disease with mediastinal and hilar lymph node enlargement (*LN* in **b**, **c**; not easily distinguished from consolidation). There is displacement of the trachea to the left and compression of the bronchus intermedius and left main bronchus (*white arrows* in **a**, **c**), and consequent distal parenchymal consolidation (*C* in **a**, **c**), lobar expansion and necrosis. CT (**c**) confirms the characteristic low-density subcarinal and right hilar enlarged lymph nodes (*LN*) on either side of the compressed bronchus intermedius (*white arrow*). A pocket of gas indicates early lung cavitation (*black arrow* in **c**)

It has been demonstrated that because infant airways are smaller and more pliable than others they can be more easily compressed by enlarged hilar lymph nodes [10]. This makes airway compression or displacement on chest radiographs an important indirect or surrogate feature of tuberculous lymphadenopathy in this age group. In the identification of airway compression, it should be remembered that normal bronchi should become of smaller caliber progressively, according to increasing distance from their origin. In trying to determine any compression of a major bronchus, comparison can also be made with a similar bronchial branching generation on the contralateral side [8]. Airway compression might represent the most objective chest radiographic sign of primary tuberculosis in children because the airway is the only discernable normal lucent structure within the confluent density of the mediastinal structures. The normal trachea in children should be to the right of the midline [8] when there is a normal left-side aortic arch. As demonstrated in Fig. 6, tuberculous mediastinal lymphadenopathy can compress and displace the trachea to the left [8] and hilar lymphadenopathy can cause compression of the bronchus intermedius and left main bronchus. The commonest site of airway compression is the bronchus intermedius, which is compressed between the right hilar lymphadenopathy and subcarinal lymphadenopathy [5].

## Normal lateral chest radiograph

**Fig. 7:**
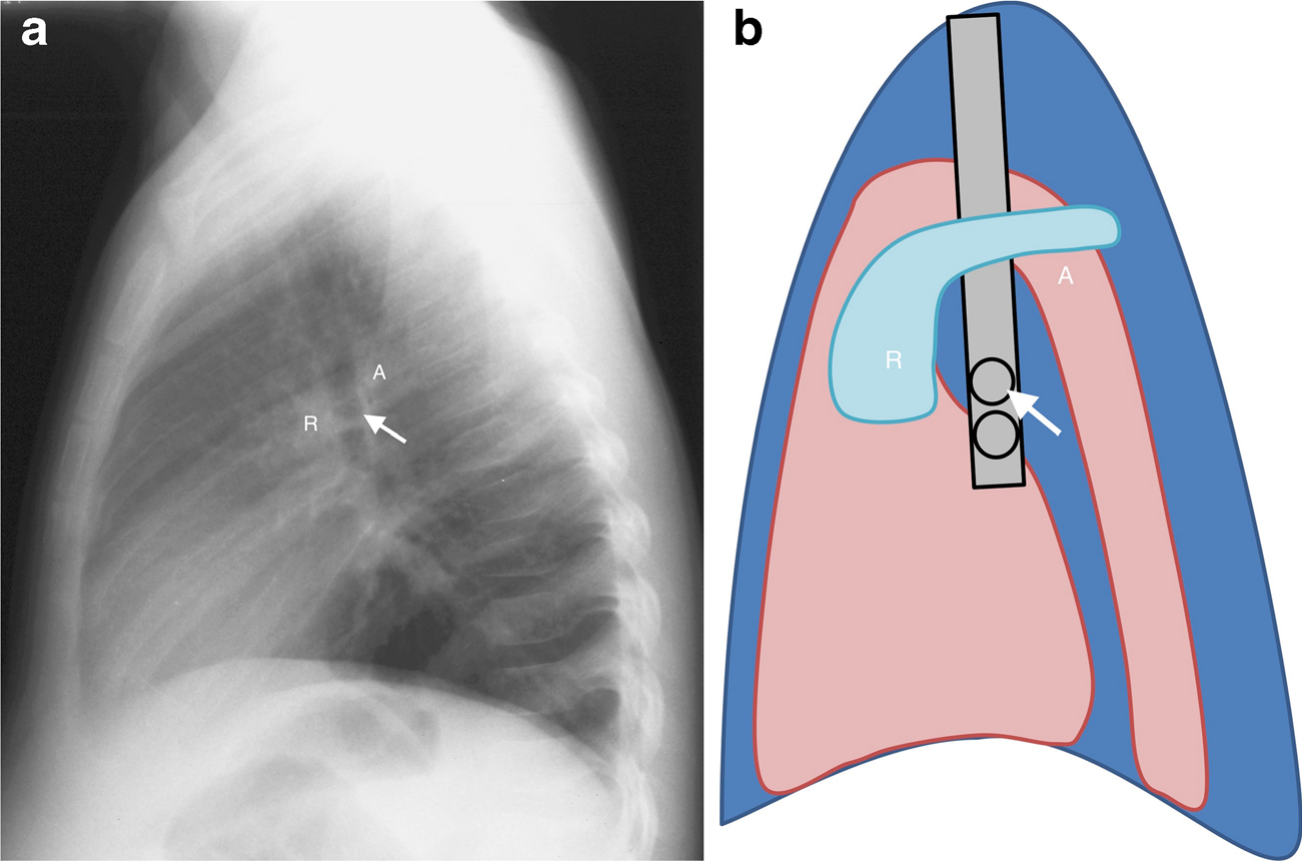
Normal lateral chest radiograph in a 6-year-old girl. **a**, **b** Lateral chest radiograph (**a**) and schematic (**b**) demonstrate the normal soft-tissue densities of the right (*R*) and left pulmonary arteries as well as the posterior aspect of the aortic arch (*A*), which form an upside-down horseshoe that is suspended at approximately the level of the carina, with only diverging linear and branching smaller vessels seen below this. The position of the carina is assumed on the lateral to be just above right upper lobe bronchus, which is the first oval lucency (*arrow*) within the lower trachea. From there the trachea continues as the bronchus intermedius. There should be no oval soft-tissue densities behind or below the carina and bronchus intermedius

Normal lateral chest radiographs should not demonstrate any oval soft-tissue densities dorsal or inferior to the lower trachea (near the carina) and bronchus intermedius. Normal soft-tissue densities of the vascular structures, the main pulmonary arteries and posterior aspect of the aortic arch should form an upside-down horseshoe above the level of the carina, while only diverging linear and branching smaller vessels should be seen below this [8] (Fig. 7). The position of the carina can be assumed when the right upper lobe bronchus is seen as an ovoid lucency within the lower trachea — from there the tracheal lucency continues as the bronchus intermedius.

## Lateral radiograph lymphadenopathy

**Fig. 8:**
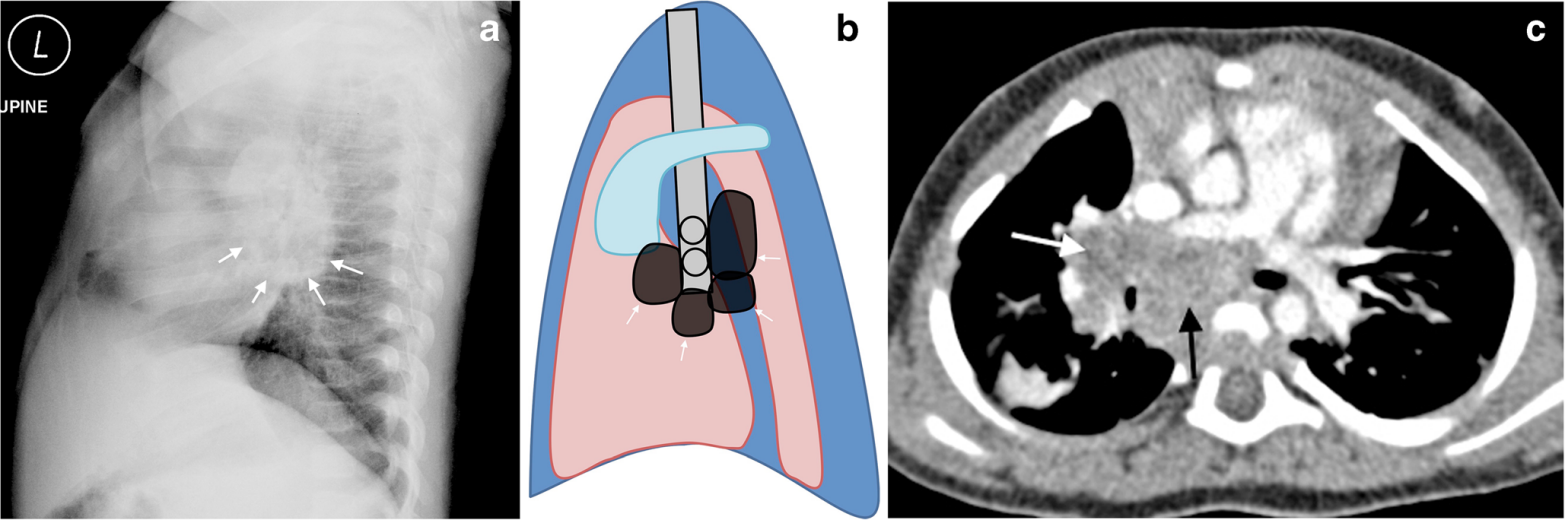
Tuberculous lymphadenopathy involving the sub-carinal and retrocarinal regions in a 4-year-old girl. **a**–**c** Lateral chest radiograph (**a**), schematic (**b**) and axial post contrast CT (**c**) demonstrate lobulated, mass-like densities posterior and inferior to the bronchus intermedius, making up the doughnut sign (*arrows* in **a** and **b**) characteristic of tuberculous lymphadenopathy. CT (**c**) confirms that the lobulated density inferior and posterior to the bronchus intermedius corresponds to subcarinal/retrocarinal enlarged lymph nodes (*black arrow* in **c**) and hilar lymphadenopathy (*white arrow* in **c**), while the upper half of the radiographic doughnut is made up of the normal right and left main pulmonary arteries and the aortic arch (not shown)

Lateral radiographs are considered useful and therefore are still obtained for detecting lymphadenopathy in children with suspected tuberculosis. Lymphadenopathy is characteristically identified as lobulated mass-like density posterior and inferior to the bronchus intermedius [2, 8]. Lymphadenopathy (inferiorly and posteriorly) combines with the densities of the normal vascular structures (superiorly) to form the inferior portion of an imagined doughnut, hence the term “doughnut sign” (Fig. 8). The lobulated density inferior and posterior to the bronchus intermedius has been shown to correspond to sub-carinal and retro-carinal lymphadenopathy by cross-sectional imaging [8] while the upper half is made up of normal soft-tissue densities of the right and left main pulmonary arteries and the aortic arch [2, 8].

## Conclusion

This pictorial review represents a comprehensive collection of chest radiographic signs of tuberculous lymphadenopathy with explanatory schematics and accompanying cross-sectional imaging confirmation, and is intended to aid clinicians who interpret chest radiographs in children with suspected pulmonary tuberculosis. It is also hoped that the publication of a pictorial standard of the appearances of obvious lymphadenopathy on chest radiographs confirmed with cross-sectional imaging in children with culture-confirmed primary pulmonary tuberculosis serves to improve interobserver agreement in the interpretation of chest radiographs for the diagnosis of pulmonary tuberculosis in children.
